# A Short Account of the Principal Medical Establishments at and near Paris; Taken Chiefly from the National Almanac of France for the 12th Year of the Republic, Ann, 1804. To Which Is Added, by Way of Comparison, a Short Sketch of Medical Lectures, and the Opportunities Afforded for Acquiring Professional Knowledge in London

**Published:** 1812-12

**Authors:** 


### *For the Medical and Physical Journal.*

### *A short* ACCOUNT *of the principal* MEDICAL ESTABLISHMENTS *at and near* PARIS; *taken chiefly from the* NATIONAL ALMANAC *of* FRANCE *for the* 12*th* YEAR *of the* REPUBLIC, *ann.* 1804. *To which is added, by way of comparison, a short* SKETCH *of* MEDICAL LECTURES, *and the opportunities afforded for acquiring* PROFESSIONAL KNOWLEDGE *in* LONDON.

IT has been asserted, that London, at this time, contains such a well-appointed scheme of medical tuition, that an additional seminary, however regulated, is wholly unnecessary and superfluous. Before we enter into any consideration of particular schools, let us contemplate the metropolis as a whole, to ascertain how far it is entitled collectively to the high encomiums which have been lavished upon it, by its zealous advocates. Of medical lecturers there is certainly no want. They are to be met with in every street; but the attending pupils confine themselves so exclusively to a few popular subjects, e. g. anatomy, surgery, and midwifery, that others of equal importance are most unaccountably overlooked.

While the mode of instruction remains on its present footing, the lectures, for want of encouragement, will never be sufficiently diversified to include a full and comprehensive system. Under such disabilities, Englishmen who aspire to embrace the healing art in its multifarious branches, must continue to emigrate into other states, to obtain, at a great expense, what could be much better provided in their own country, were its native mines properly explored. The truth of these remarks will be rendered strikingly obvious, on comparing the medical lectures in Paris with those of London.

1st. THE MEDICAL SCHOOL at PARIS, founded since the revolution, contains the following courses:—Anatomy and Physiology, by Chaussier and Dumeril.—Medical Chemistry and Pharmacy, by Fourcroy and Deyeux.—Practice of Medicine and Dietetics, by Halli and Desgenettes.—External Pathology, by Lassus and Percy.—Internal Pathology, by

Pinel

Pinel and Bourdier.—Botany and Natural History with reference to Medicine, by Peyrilhe and Richard.—Surgical Operations, by Sabatier and Lallement.—External Diseases requiring attendance at the bed-side, by Pelletan and Boyer. —Internal Diseases requiring attendance at the bed-side, by Corvisart and Le Roux de Tillets.—Rules for the Conduct of Physicians, particularly with regard to their behaviour in the sick room, by Dubois and Petit Radel.—Midwifery, by Alphonse Le Roy and Baudelocqué.—Medical Jurisprudence, by Leclerc and Cabanis.—The Doctrines of Hippocrates and on rare cases, by Thouret.

*Medical Bookseller*—Sue.

Demonstration of Drugs and of Surgical Instruments, by Thillaye.

*Artists attached to the School*—Dupreytren, chief of the anatomical department; Lomonnier, painter; Pinson, modeller in wax.

2d. School of Rural Economy, and of Veterinary Medicine, situated at d'Alfort.

This establishment, founded in 1765, by Bourgelat, was confirmed by law in the third year of the republic, anno 1805. The minister of the interior has it under his department. It contains a library, a cabinet of comparative anatomy, and another for pathology. They are open to the public every day. There are also large and well-appointed hospitals for the treatment of diseased animals; forges; chemical and pharmaceutical laboratories; a botanic garden; land for growing fodder; bee-hives; a flock of sheep, intended for experiments in mixing the breeds, and in improving the wool; a great room for lectures, and apartments for lodging and employing the pupils. When epizootic maladies afflict the provinces, one or more pupils, and sometimes the professors, are sent down to treat the distempered animals, for which they charge only the expenses incurred.

*Professors*—The Anatomy and Physiology of all domestic Animals, are taught by Girard.—Rules for choosing Horses, Asses, and Mules; general Dietetics; the construction of Apartments, and mode of Lodging; are given by Godine.—Dietetics applied to the rearing of other domestic Animals, their Choice; a particular School for Shepherds; and the Library; are under the direction of Godine, junior.—Botany, Pharmaceutical Chemistry, Materia Medica, &c. by Dapuy. —The Theory of Diseases, Surgery, Jurisprudence, and Farriery, by Fromage.—Attendance upon disordered Animals in and out of the school, by Chaumontel.

*Private Teachers*—Le Villain, Cretiennot, Godard, Meurier, Herseut, Thuillier.

*Jury*

*Jury of Veterinary Examiners*—Huzard, Doublet, Cesar, and Desplato.

*Jury of Examiners in Agriculture*—Parmentier, Texier, Yvart, and Celo.

It is understood that this institution has been lately removed to Paris, for the mutual accommodation of medical and veterinary students; and that various important additions have been made to both departments of the curative art.

3d. THE NATIONAL MUSEUM of NATURAL HISTORY constitutes a branch of useful knowledge to the accomplished physician, who, as the follower of nature, ought to be familiarly acquainted with her diversified productions. It contains a botanic garden, a collection of natural history, a room for lectures, an appropriate library, and a menagerie for living animals. The gallery of natural history, and library, are open every day to strangers or students. The garden supplies the provinces with seeds, trees, and plants, serviceable to agriculture and the arts: it also furnishes the sick poor with articles for the cure and relief of their several disorders.

The professors and directors of this splendid and truly-important establishment are, Fourcroy, for general chemistry; Brongniart, for the chemical arts; Desfontaines has the direction of general botany; Jussieu conducts the rural department; Geoffroy has the care of the zoology of quadrupeds, whales, and birds; Lacepede those of reptiles and fish; Lamarck the zoology of insects, and worms comprised under the denomination of shell-fish, zoophites, litophites, and animalcules, requiring microscopic aid; Portal teaches human anatomy; Cuvier that of animals; Hauy is appointed to mineralogy; Andre Thouin to the cultivation of the gardens; Foujas has the care of geology; and Vanspaendonck makes drawings of curious and rare specimens.

There are also librarians, gardeners, curators, secretaries, &c. besides assistant naturalists. The free-school of pharmacy includes a garden for medicinal herbs, and a pharmaceutical laboratory, where apothecaries are more particularly instructed. At the military hospital, the lectures, and medical treatment, are chiefly adapted to the education of medical health-officers intended for the army or navy.

Certificates are indispensable preliminaries to practise, and are always given after strict public examinations, which are partly in the Latin, partly in the French, languages.

Such were the opportunities afforded at Paris, and the state of medical tuition, so long ago as the twelfth year of the French Republic. That they have undergone many subsequent improvements, under the immediate direction of the present chief of the empire, is well known, though we are not not sufficiently acquainted with their nature and extent to detail them with the requisite precision.

In one part or other of the British metropolis, we have, it is true, instructions in anatomy, surgery, physiology, theory and practice of medicine, midwifery, chemistry, botany, materia medica, clinical lectures, diseases of the teeth, comparative anatomy, and the complaints of horses.

Such is the boasted programma of London. Compared with Paris, it makes only a poor display, even if we should admit, which is certainly not true, that all the courses are comprehensive and ably delivered. As the metropolis may be said to include four or five distinct and independent schools, these several branches are no where taught in methodical connexion, or from the vast extent of the capital to be conveniently approached from any fixed point. This is a great defect, since it necessarily occasions much loss of valuable time, and exposes the inexperienced to dangerous allurements in their progress through the streets.

2d. Suppose a school to be opened, according to the Parisian plan, but conducted as at present, by unauthorised professors, it would be highly defective in essential particulars. In London two courses are repeated in the short space of six or seven months, which makes it impossible for the teachers to exhibit more than a smattering of any branch of the profession. In Edinburgh a single term employs nearly the same period, while in Paris each course is reported to occupy full ten months. Here then we discover a striking difference in the medical arrangements of the two countries, highly unfavorable to our own, where a general and superficial plan of tuition is alone attainable. This process, however long continued, is quite insufficient to furnish all that is wanted, in any department of medical science, so that a London education is necessarily inadequate to the end proposed.

3d. Were the metropolis to be put upon the best possible regulation in these respects, the omission of examinations to incite the studious to further exertions, and distinguish meritorious individuals from lazy and careless drones, would prove an inherent defect, tending to repress aspiring genius, and to place every practitioner on the same level, the moment he entered upon medical competition.

4th. This want of discrimination is most felt in the certificates of attendance. They are always made to recommend the possessor for regularity, attention, and diligence, even when it had been notoriously observed that he was negligent, disorderly, and generally out of the way. It has been openly asserted by persons of undoubted veracity, that some some celebrated living professors may be named, who have not objected to attest for several courses of lectures, when only one had been attended, and that imperfectly. Where tickets for lectures have been taken out and paid for, the certificates follow in course, even although the applicant had been absent in a distant part of the kingdom for nearly the whole term. Inquiries are perhaps never made with a view to regulate the language of these virtual diplomas, or fix the wavering determination of the grantor. A London tradesman and his wife, in passing through a provincial town, read the name of a person, in large characters, with surgeon, apothecary, and man-midwife, subjoined, in gilded letters. Suspecting the advertiser to have lately been a menial servant of theirs, and wholly unacquainted with medicine, the husband resolved to satisfy the curiosity excited. He accordingly returned the following morning to investigate the affair. The object of research being speedily rencontred in the open street, all doubt of his identity was removed, by an inquiry after his old mistress. This led to an interrogation, whether the gilded sign belonged to him. "O, yes!" was the answer: "accompany me home, and you shall see my credentials." The invitation being readily accepted, a long list of certificates for courses of lectures in anatomy, surgery, midwifery, chemistry, the theory and practice of medicine, &c. &c. was discovered hung up in the surgery, splendidly framed and glazed, to astonish the inconsiderate multitude, and remove all doubts of the possessor's unerring attainments. Still incredulous, our cautious traveller noted what he saw. He ascertained that all the tickets had been obtained in one day, and that the certificates bore an uniform date. Having taken leave, he hastened in amazement to the house of a medical friend, to communicate what he had witnessed. Here the veil was soon removed. The quondam footman, it was ascertained, came down fresh from the tradesman's service, to encounter all the perplexities of medical practice, though he had only been conversant with the menial employment of a barber-surgeon, in which he was tolerably expert.

After remaining some time, he privately withdrew himself for nearly three weeks, and then returned to the old quarters, where he had continued ever since selling drugs, and interfering in all ailments internal and external. During this short absence, he had taken out the above-mentioned tickets from the most eminent London preceptors, and obtained afterwards the usual certificates of diligent attendance, confirmed by the approving signatures of the respective masters. —Such are the nefarious means used to conciliate the good opinion of patients, and impose upon a credulous people. —Another glaring incident of the same kind remains to be added. The usual certificates were given in this instance to a drug-selling grocer, during the progress of the course, and after a still shorter residence in London.—Were more testimonies wanted, every county, perhaps every considerable town, might be made to furnish a living proof of the same kind. Incredible as it may appear to the untutored reader, that men of great professional celebrity, and of otherwise unblemished reputation, should humble themselves to such low and pitiful expedients to get pupils; the fact is too well known to admit of controversion. This mode, as dangerous to the community as it is discreditable to the parties themselves, can never be effectually checked until both teachers and pupils become subject to an impartial tribunal.

Such is London at this time, a city stored with the choicest materials, and the most neglected. Look at the streets, perpetually crouded with an exuberant population, and the spacious docks charged with vessels from every port. Her hospitals and charities are the most splendid and most numerous, proclaiming to all nations the wealth and the benevolence of Englishmen. And shall these opportunities for training medical candidates, unequalled in extent and variety, be for ever overlooked? No! let them be industriously collected, and arranged in some convenient spot, where systematic education may be conducted on a plan as superior as the means are ample. Let it include full instructions for army and navy cadets, that our brave defenders may in every climate be preserved from the ravages of disease, to hurl the British thunder upon her inveterate foes. Let it also comprehend the maladies of animals and of plants, that all living nature may be brought forward, mutually to illustrate and explain. It is in this way that the minds of youth destined to practise in any department of the curative art should be formed and trained. Thus stored they will be prepared to improve and enrich the particular line in which they are destined to move. Then shall we see physicians, veterinary practitioners, and agricultors, conversing freely on the same topics, and imparting knowledge by their particular observations. In this way a generous emulation and rivalship will be created, highly advantageous to medical science, and the best interests of mankind. This plan, destined to exalt the curative art in England above foreign competition, should in no wise interfere with private teaching. The lecturers who refuse to embark in the new scheme may continue, as at this time, private competitors; but let there be one place in the capital where examinations are indispensable, and certificates certificates are honestly conferred. Under such an arrangement, an additional seminary will, it is true, be founded in the metropolis; but, like the rest, it must owe its celebrity to the abilities and diligence displayed, and opportunities afforded.

In France, the most powerful ministers have been desirous to encourage the literary establishments of the empire. The Veterinary College at d'Altfort, was founded by the great Bourgelat, who confided it to the particular direction and superintendence of the minister of the interior. Surrounding nations soon discovered its value, and sent pupils thither to learn the veterinary art. On their return home, similar institutions were erected. There is scarcely a state on the continent of Europe which has not its academy for teaching rural economy and the veterinary art. By these wise provisions, the produce of the earth, and the health of animals, have been greatly promoted. Splendid establishments for the study of natural history, and general science, have also been founded at Paris, and brought to perfection by successive ministers. The Medical School, erected, upon a magnificent plan, since the revolution, has been an object of unceasing anxiety to the governing authorities, and especially to the present ruler, who has publicly declared, that the successful issue of several campaigns, and of his greatest victories, were to be imputed as much to the skill and attention of the health officers, as to the superior tactics and discipline of his army.

Happy will it be for the British empire, when some great and good man, possessing the power and the inclination to confer a real blessing upon his fellow creatures, shall employ his abilities to draw out the unparalleled resources of this opulent nation, for the general improvement of medical knowledge and practice at London. His name, endeared to his country, will be transmitted to the remotest ages as a friend to humanity, and the patron of useful learning.

